# The Macroeconomic Consequences of Renouncing to Universal Access to Antiretroviral Treatment for HIV in Africa: A Micro-Simulation Model

**DOI:** 10.1371/journal.pone.0034101

**Published:** 2012-04-13

**Authors:** Bruno Ventelou, Yves Arrighi, Robert Greener, Erik Lamontagne, Patrizia Carrieri, Jean-Paul Moatti

**Affiliations:** 1 INSERM, U912 (SESSTIM), Marseille, France; 2 Aix-Marseille School of Economics – CNRS – Greqam, Marseille, France; 3 The Regional Health Observatory of Provence-Alpes-Cote d'Azur, Marseille, France; 4 The Joint United Nations Programme on HIV and AIDS, Geneva, Switzerland; 5 Aix-Marseille Univ, IRD, UMR-S912, Marseille, France; University of Calgary, Canada

## Abstract

**Aim:**

Previous economic literature on the cost-effectiveness of antiretroviral treatment (ART) programs has been mainly focused on the microeconomic consequences of alternative use of resources devoted to the fight against the HIV pandemic. We rather aim at forecasting the consequences of alternative scenarios for the macroeconomic performance of countries.

**Methods:**

We used a micro-simulation model based on individuals aged 15–49 selected from nationally representative surveys (*DHS* for Cameroon, Tanzania and Swaziland) to compare alternative scenarios : 1-freezing of ART programs to current levels of access, 2- universal access (scaling up to 100% coverage by 2015, with two variants defining ART eligibility according to previous or current WHO guidelines). We introduced an “artificial” ageing process by programming methods. Individuals could evolve through different health states: HIV negative, HIV positive (with different stages of the syndrome). Scenarios of ART procurement determine this dynamics. The macroeconomic impact is obtained using sample weights that take into account the resulting age-structure of the population in each scenario and modeling of the consequences on total growth of the economy.

**Results:**

Increased levels of ART coverage result in decreasing HIV incidence and related mortality. Universal access to ART has a positive impact on workers' productivity; the evaluations performed for Swaziland and Cameroon show that universal access would imply net cost-savings at the scale of the society, when the full macroeconomic consequences are introduced in the calculations. In Tanzania, ART access programs imply a net cost for the economy, but 70% of costs are covered by GDP gains at the 2034 horizon, even in the extended coverage option promoted by WHO guidelines initiating ART at levels of 350 cc/mm^3^ CD4 cell counts.

**Conclusion:**

Universal Access ART scaling-up strategies, which are more costly in the short term, remain the best economic choice in the long term. Renouncing or significantly delaying the achievement of this goal, due to “legitimate” short term budgetary constraints would be a misguided choice.

## Introduction

In its strategy for 2011–2015, UNAIDS states that “a renewed advocacy effort must be launched to encourage the continued commitment of the global North to support development efforts in the global South, with a focus on long-term predictable financing, particularly through multilateral mechanisms” [1]. Indeed following the 2001 Declaration of Commitment of the United Nations General Assembly Special Session on HIV/AIDS (UNGASS) and its further 2006 recommendation to scale up services and interventions “towards the goal of providing universal access to HIV prevention, treatment and care by 2015”, global funding for HIV programs has spectacularly increased from US$ 1.4 billion in 2000 to 15.6 billion in 2009. It is estimated that approximately 70% of total spending for HIV in low and middle-income countries comes in the form of international assistance [2]. Significant advances in the fight against the pandemic have been obtained from these efforts. Globally, HIV infections are fewer now than ten years ago, reflecting several factors that include the natural course of the epidemic and the impact of HIV prevention efforts. By the end of 2009, over five million people in low- and middle-income countries were reported to be receiving antiretroviral treatment (ART), including eight countries providing ART to at least 80% of patients in need and 21 additional ones with coverage rates higher than 50%, an achievement that would not have been deemed possible five years ago [3]. Progress has been most noticeable in sub-Saharan Africa the world's region most hardly hit by the epidemic [4].

In the context of the worst financial and economic crisis since the 1930's, there are growing concerns that global HIV funding will be flat-lined or even reduced in the next future [5]. Short-term sustainability of scaling up ART coverage to reach universal access is particularly under question since WHO 2010 recommendations to initiate ART at an earlier stage of the disease have mechanically increased the number of people eligible for treatment from an estimated 10.1 million to 14.6 million worldwide [6], and since growing number of patients are in need of more expensive new first-line and second-line antiretroviral regimens [7]. Pledges by governments and private donors for the 2011–2013 third voluntary replenishment of the Global Fund to Fight AIDS, Tuberculosis and Malaria (GFATM), although amounting to US$ 11.7 billion, fell short of the minimum target set by the GFATM (US$ 13 billion) and from its own needs estimates to keep on track with the goal of universal access in 2015 (US$ 18–20 billion) [8]. As acknowledged by the Executive Director of GFATM, “the replenishment will enable further significant scale up, but not at the same pace as in recent years and is insufficient to meet anticipated demand” [9]. Moreover, the fiscal-year 2011 budget of the US President's Emergency Plan for AIDS Relief (PEPfAR), the other major donor for funding of ART, proposes only a 2% increase, which may result in future treatment enrollment freezes [10]. Indeed, the long-term sustainability and economic rationale of scaling up access to ART is increasingly questioned in both academic and policy circles [11]. Exploring the macroeconomic impact on GDP growth and human development of renouncing to -or delaying- the goal of universal access to HIV care and treatment is therefore key for “making sense of the money” devoted to HIV by bilateral and multilateral donor programs [12].

In previous economic literature, there have been a number of exercises aimed at estimating future funding needs for scaling up HIV programs in low and middle income countries based on extrapolation of current expenditures and epidemiologic modeling [13], [14]. On the other hand, there have been macroeconomic estimations of the impact of HIV/AIDS integrating epidemiological dynamics into neoclassical or “endogenous growth” models [15]–[18]. However, proper estimates of the economic impact of alternative funding scenarios for HIV treatment have to be based on the potential consequences of each scenario for the future life-history of affected individuals and, jointly, on an aggregation of these effects for the whole dynamics of the economy. This is now made possible by the availability of individual data on socio-economic characteristics and health-seeking behaviors of nationally representative population samples through Demographic and Health Surveys (DHS) and the use of dynamic micro-simulation methods[19]. To our knowledge, this paper is the first attempt to compare the long term (mid 2030's) economic consequences of two archetypal “extreme” scenarios for funding access to ART: the UNGASS inspired universal access scenario (scaling-up to 100% coverage by 2015, with two variants defining ART eligibility according to previous or current WHO guidelines), *versus* a “freezing” scenario in which the currently observed ART coverage is maintained without further increase in ART provision. This modeling exercise was carried out in the case of three African countries where high-quality DHS were available and which differ in terms of HIV prevalence and current level of wealth: Cameroon, Tanzania and Swaziland. Per capita Gross Domestic Product (GDP) ranges from US$ 347 in Tanzania and US$ 906 in Cameroon, to US$ 2659 for Swaziland [20]. In Cameroon and Tanzania HIV prevalence rates are relatively high but lower than Swaziland where it is one of the highest in the world [2].

## Materials and Methods

### Overview

To estimate the future impact of the HIV epidemic under different levels of ART procurement in Cameroon, Swaziland and Tanzania, the following steps were carried out.

1. We design a micro-simulation model based on individuals aged 15–49 selected from nationally representative surveys. By this process each individual may be seen as “representative” of a portion of the general population of the country.
2. We introduced an “artificial” ageing process, by computer programming methods. Individuals could evolve through four different health states: HIV negative, HIV positive (with two stages of the disease) and death. Four ART procurement scenarios determine these dynamics through their consequences on survival rates of the affected population and on infection rates in the general population: No Access (*S0*), Aid Freeze (*S1*), Universal Access (all patients with CD4 cell counts <200/µl)(*S2a*), and Extended Universal Access (all patients with CD4 cell counts <350/µl) (*S2b*).
3. At the end of the artificial ageing process, we created a “picture” for each given country in the mid-2030's not only in terms of ART need and procurement, lives saved, and HIV infections prevented (compared to *S0*), but also in terms of macroeconomic aggregates, derived for each scenario (costs and economic benefits). The macroeconomic impact is obtained using sample weights that take into account the resulting age-structure of the population in each scenario.

### Datasources: Representative Agents

The simulations of this paper are based on three databases from the worldwide MEASURE Demographic and Health Surveys (DHS) program: the Tanzania HIV/AIDS Indicator Survey 2003–04 (THIS), the Cameroon Demographic and Health Survey 2004 (EDSC), and the Swaziland Demographic and Health Survey 2006–07 (SDHS) [21]–[23].THIS, SDHS and EDSC are the first nationwide surveys to provide HIV prevalence estimates: in addition to the data collected through interviews, respondents aged 15–49 were asked to voluntary provide a blood sample for subsequent HIV testing (regardless the severity of HIV). The HIV testing protocols have been approved by ORC Macro ethical comity. They were based on the anonymous linked protocol developed by DHS, which allows for the linking of the HIV test results to the socio-demographic data, provided that information potentially identifying an individual is destroyed before the linking takes place. Among all respondents, 80.5% in Tanzania (*TZ*), 82.7% in Swaziland (SZ) and 91.0% in Cameroon (*CM*) agreed to participate to the HIV testing protocol. The sampling procedure was determined before HIV testing agreement in order to avoid biases in the representativeness of the general population. Besides, non-participation to the HIV testing protocol has been shown to have negligible bias on HIV prevalence estimates [21]–[23].The final samples for the simulations comprised 10,747 individuals in *TZ*, 8,187 observations in SZ and 9,751 records in *CM*, characterized by gender, 5 years age group and HIV status (divided into two stages, asymptomatic and symptomatic; see Appendix S1 for methodology).

### Ageing Process: A Micro-Simulation Model

**Figure 1:**
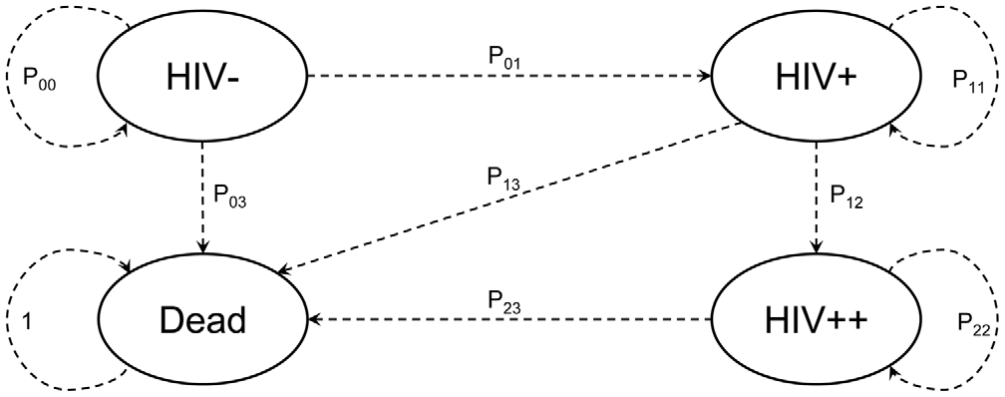
State transition diagram of the Markov chain. Ellipses define the four different health states of the model (HIV−, HIV+, HIV++ and Death). The dashed arrows represent the probabilities of going from one state to another in the subsequent period.

An individual's future health status was forecasted using a discrete-time micro-simulation model. In the model, an individual is, at a given period, either HIV negative (HIV−) in the asymptomatic stage of HIV (HIV+), in the symptomatic stage of HIV (HIV++) or deceased (D). At a time *t*, an individual is described by his/her health status. At the next period *t+1* (one period lasting 5 years), the same person's health status is determined by a transition rate matrix –age and gender specific- according to his/her previous health status. This Markovian process can be summarized by Figure 1. We opted for five years periods in the model shorter intervals (e.g. one year).Indeed, DHS exhibit considerable sampling variations and non-robustness which could lead to spurious annual transition rates. It would follow that results at the 2040 horizon, resulting from 30 simulation cycles, may be severely biased.

Appendix S1 describes how we compute each transition probability P_k;j_ (the probabilities of going from HIV− to HIV−(P_00_), from HIV− to HIV+ (P_01_), etc). HIV being a chronic and “progressive” disease, the probabilities of going from HIV+ to HIV− (P_10_), from HIV++ to HIV− (P_20_) and from HIV++ to HIV+ (P_21_) are null. P_33_is equal to 1 (death being an absorbing state).

An essential characteristic of our micro modelling is that this set of probabilities changes along with the policy scenario (e.g., P_23_, probability from HIV++ to death, is reduced in the case of access to treatment). They are computed on the basis of DHS surveys and litterature estimates (e.g. WHO/UNAIDS estimates of the HIV epidemic[2]; refer to Appendix S1 for details on the methodology).

During the simulation process, individuals becoming older than 54 are removed from the simulation sample (i.e. those aged 45–49 in the initial period are censored after the second period, those aged 40–44 in the first period are censored after the third period,…), as we cannot establish transition probabilities nor the future of these age groups. When the “oldest” cohort (aged 50–54 in *t*) is censored in *t+5* it is automatically replaced by the preceding cohort (aged 45-49 in *t*, becoming 50–54 in *t+5*). This issue is similar for the youngest cohort (aged 15–19 in *t*, becoming 20–24 in *t+5*). A new cohort of individuals aged 15–19, taken from the original DHS dataset, is introduced at every time period in the simulation sample to mimic real-life population dynamics. In order to take into account the demographic evolution, the 15–19 population's five-year growth rate is estimated and allows us to include a new “demographic-adjusted size” cohort at each period (see Table 1 for values) [24].

**Table 1:** Model Key Parameters.

Parameter			Swaziland	Tanzania	Cameroon
**Demographic**	*15–-24 Population*	*Growth Rate*	14.18%	13.56%	11.71%
	*15–-49 Population*	*(In Thousands)*	582	16,220	7,708
	*Sample Weights*		71	1,521	790
	*Sample Sizes*		8,187	10,747	9,751
**Economics**	*Employment rates:*	*Age [15–-19]*	Reference		
	*Estimated*	*Age [20–-24]*	1.538	1.339	1.299
	*Coefficients from the*	*Age [25–-29]*	2.378	2.284	2.025
	*Logit Model*	*Age [30–-34]*	2.649	2.514	2.618
		*Age * [*35]*–[39**]	2.836	2.702	2.929
		*Age * [*40]*–[44**]	2.696	2.934	3.094
		*Age * [*45]*–[49**]	2.691	2.677	2.977
		*Woman*	−0.7567	-0.369	−0.596
		*Intercept*	−1.580	0.196	−0.663
	*Absenteeism Rates*	*HIV −*	100%		
		*HIV+*	100%		
		*HIV++ w/ ART*	95%		
		*HIV++ w/o ART*	75%		
	*GDP at Market Price*	*In billions USD*	2.648	11.351	15.775
	*Annual Avg. Wage*	*Per Worker*	$10,381	$870	$3,223
**Epidemiology**	τ *(ART need; 2006 Guidelines )*		31.05%	30.00%	29.91%
	TC *(ART coverage; 2006 Guidelines )*	*T0 (Initial Stage)*	42.37%	0.71%	8.75%
		*T0-T1*	Unknown	16%	18%
		*T1 (First Period)*	Unknown	31%	26%
	*α (HIV history)*		0.1		
	v *(HIV+ Mortality Gap)*		0.005		
	u *(HIV++ Mortality Gap)*		0.2 for *S1* & *S2a* ; 0.1 for *S2b*		
	γ *( HIV Diffusion Parameter)*		0.518	0.388	0.511
	ϕ *(Prevention Parameter)*		0 for *S1* & *S2a* ; 0.7 for *S2b*		
	*β (Transition Booster)*		1.30	1.27	1.30
	ψ (*Transition Restrainer*)		0.7 for asymptomatic patients ; 1 otherwise		

### Microsimulating the impact of ART Procurement Strategies

Micro-simulation methods allowed us to compare two ARV access scenarios: “Aid Freeze” (*S1*) and “Universal Access” (*S2*), the latter being broken down into two scenarios -whether 2006 (for *S2a*) or 2010 (for *S2b*, “Extended Universal Access”) ART eligibility criteria are considered [25], [26]. The WHO recommended in 2006 that HIV infected people with a CD4 cell count ≤200 cells/µl, those in clinical stage III with a CD4 cell count ≤350 cells/µl and those with a diagnosis of WHO stage IV disease should start ART [25]. Since 2010 the WHO recommends that people with a CD4 cell count ≤350 cells/µl should start ART regardless of the presence or absence of clinical symptoms and that those with a diagnosis of WHO stage III or IV should start ART irrespective of their CD4 cell count [26]. These scenarios are contrasted between each other, but also versus a baseline scenario (“No Access”, *S0*) which provides a picture of what the world would have looked like if ARVs procurement had not been introduced in the three studied countries and remained null during the whole period. “No-Access” and “Universal Access” scenarios can be considered as hypothetical boundary scenarios: the “Universal Access” scenarios –i.e. all those who need ART have access to it- show what could happen if the scaling-up of ART program was achieved in 2015. In *S2b*, the target population is 1.49 times greater than in *S2a* [27]: in addition to every HIV++ individual under ART in *S2a*, an additional subset of the HIV+ population with higher CD4 cell counts (200<CD4<350) receives ARVs. The “Aid-Freeze” scenario represents what could be the extreme consequences of renouncing to further ART scale up due to the budgetary constraints of the financial crisis: the current number of PLWHIV receiving ARVs remains constant (i.e. a “freeze” in the absolute number of ARV treatments delivered).

Thanks to the explicit modeling of individual ageing we can include the consequences of each scenario for the future life-history of individuals. Roughly speaking, we deform the transition matrix in order to capture the main effects of a program. HIV++ individuals live longer when receiving ARVs, and the earlier they start ART, the higher their survival rate is (see Appendix S1 and Table 1). Moreover ARVs do not only reduce mortality: when a large proportion of the infected population is treated, as in scenarios *S2a* and *S2b*, ART is also thought to have a preventative effect, in terms of an individual's infectiousness, which is negatively correlated to the delay between HIV infection and ART initiation [28]. This reduction in HIV−transmission rates (ϕ, see Appendix S1) is thus assumed to be higher in *S2b* than in *S2a*. For instance, the baseline infection probabilities are multiplied by one in *S2a* (a conservative assumption) and by 0.3 in *S2b* (a rather optimistic hypothesis; Table 1). The outcomes' sensitivity to these assumptions will also be assessed.

### Economic assumptions

When assessing the cost-benefit of a healthcare program, the costs and benefits components must be determined. The latter component can include two aspects: the gains in the economic production and those attached with better health outcomes. This paper being driven by an accounting approach, we only measure the economic benefit as production gains.

DHS include a module on respondents' employment status: male and female aged 15 and above were asked whether they were working in the 7 days preceding the interview. We propose to estimate probabilities of participating to the labor market and to integrate this dynamic behavior into the micro-simulation model. Using a binary Logit model, labor participation is explained by the respondent's age group and gender (Table 1). Employment rate is a concave function of age and is higher among men than among women. Several authors examined the impact of HIV on economic activity in terms of productivity loss [29]–[32]. Regarding these works, we assume that compared to HIV− workers (exhibiting a null absenteeism rate), HIV+ individuals (asymptomatic stage) are not statistically more absent, while HIV++ (symptomatic without ART) workers are 25% more absent (but solely 5% more absent with ART; Table 1). Although the impact of HIV on productivity may be more severe as individuals get older, the model's absenteeism rates were assumed age-invariant due to the lack of appropriate calibration data. The age-gender specific employment rates were then multiplied by health condition-specific presence-at-work rates to obtain individual productivity rates.

We also estimate country-specific maximal average incomes per working adult (at full productivity rate) by dividing the national GDP by the number of working adults aged 15–49 in the country (Table 1) [20], [24]. The product between productivity rates & potential income provides a vector of age/gender & health condition specific wages that reproduce GDP per capita national levels. Aggregate GDP levels were computed by aggregating individual wages (multiplied by the representative weight of each observation, i.e. the number of “real” agents one DHS interviewee represents). GDP dynamics only result in the microsimulation model from epidemiologic and demographic changes: no exogenous GDP growth rate is included (a conservative assumption).

In order to compute program costs, we assume for all three scenarios that patients receive first-line ARVs for five years (210$ per patient per year) before receiving second-line ARVs until their death (590$ per patient per year) [33], [34]. In developing countries, 1.9% of 1^st^ line drugs patients switch to 2^nd^ line regimens each year [35]. Thus the model's regimens switching rate is probably overestimated, even for the future (after 2015). However, this assumption is voluntary conservative: the lower the switching rate, the lower programs associated costs would be. Laboratory tests (191$ per patient per year) and service delivery costs (72$) were also included in the costs component [34]. We do not make any additional hypothesis about ART characteristics in the future e.g. price variations, innovative or more effective treatment strategies, etc. Yet, the decreasing trend of ARVs prices suggests that our cost data may over-estimate future costs [33], [36]. The total cost of each scenario can be computed by aggregating individual costs and will be used for the macro-economic evaluation.

### Health and macroeconomic outcomes

The micro-simulation model offers a large panel of outcomes, providing public health and economic “snapshots” for each country and each five-year period: in addition to several epidemiological indicators (number of deaths averted, HIV prevalent and incident cases (and prevented cases), and treatment need; see Table 2), program characteristics and associated economic outcomes were estimated for the alternative scenarios (volume of ARV procurement, programs' costs and economic benefits; see Table 3). Time series of GDP and GDP gains (relative to *S0*) are computed for each alternative scenario by using standard aggregations methods throughout the surviving agents (the age-structure of the population and the total labor supply are endogenously determined by the aging process).

In order to compare the ART access scenarios with the counterfactual *S0* (no access to ART) in terms of economic sustainability, the common indicator “self-financing ratio” (SFR) was computed –the quotient between the total economic gains generated by treating HIV-infected workers and its associated costs. Therefore, a SFR superior to 1 means that the HIV program is cost-saving: its net contribution to the economy is positive because its global macroeconomic benefit is superior to its total costs. Alternatively, a SFR inferior to 1 means the program has a net cost for the economy but this does not necessarily means it is not worthwhile since this net cost translates, beyond its macroeconomic impact on GDP, in welfare improvements due to increase in life expectancy and quality of life of the HIV-infected population.

## Results

### Microsimulation of life paths: an illustration

**Figure 2:**
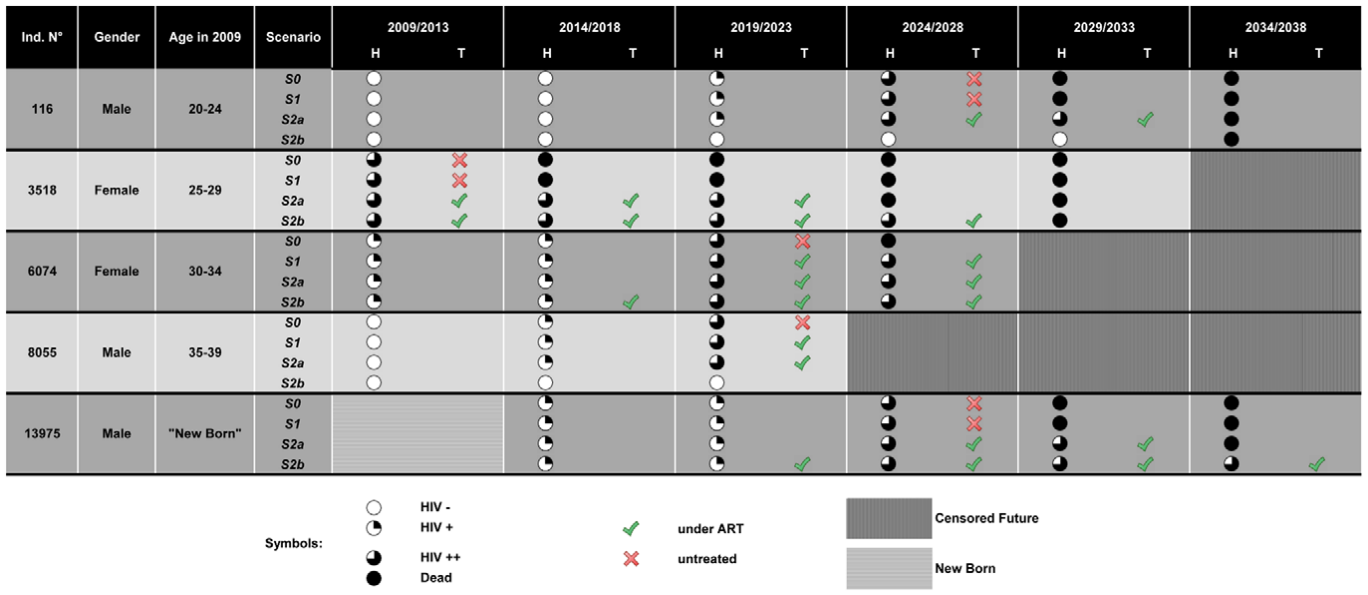
Life Paths. Simulated health trajectories of a THIS 03–04 subsample as per the four ART procurement scenarios. In columns 2009/13,…,2034/38, the full-white circles refer to individuals being HIV negative at a given period, the one-quarter black circles to HIV+, the three-quarter black circles to HIV++ and the full-black circles to deceased individuals. The ART circles columns provide information on whether or not individuals are treated with ARVs.

Figure 2 presents health trajectories from a THIS 2003–04 subsample according to the four alternative scenarios. This ageing process creates one possible future from many others for every DHS representative agent (the Markov process is a random process). Individual No. 116 is a man aged between 20 and 24 years in 2009; he contracts HIV in 2019 in scenarios *S0*, *S1* & *S2a*. He progresses to advanced HIV in 2024 and receives ARVs solely in *S2a* (he lives one more period than in *S0* & *S1*). If the *S2b* program had been set up, he would never have contracted HIV (preventative effect of ART ϕ > 0) and would have died in 2034 from other causes than HIV. Individual No. 3,518 is a HIV++ woman in 2009. She dies in the subsequent period in *S0* & *S1.* With *S2a* & *S2b* she remains under ART until her death (due to ART failure or external causes), respectively in 2024 and 2029. Individual No. 6,074 is HIV+ at the initial stage. She meets 2010 eligibility criteria in the 2^nd^ period and starts ART in *S2b*. In the subsequent period she shifts to HIV++ and obtains ART in *S2a,* before exiting from the observation window (we could not establish transition probabilities for individuals older than 54). Individual No. 8,055 contracts HIV between 2010 and 2014 in all scenarios except *S2b* and then receives ARVs in *S1* & *S2a* before being censored; his observed survival is equal in all scenarios. Individual No. 13,975 is introduced in the simulation process in 2014. He starts ART earlier in *S2b* than in *S2a* and thus lives longer in *S2b* (lower mortality with ART when initiated early). The epidemiological results at national levels are based on the aggregation of all individual simulated life paths.

### Epidemiologic Impact

As shown in Table 2, not having provided ARVs (*S0*) would have had a catastrophic impact on life expectancy through HIV-related mortality: in Swaziland (*SZ*), 439,000 individuals would die during the course of the simulation, 83% of them being HIV infected at the time of death. Tanzania (*TZ*) would experience 3.1 million deaths over 25 years and Cameroon (*CM*) 1.8 million. Compared with the absence of ART (*S0*), extended Universal Access (*S2b*) is the scenario that saves the greatest number of lives (238,000 in *SZ* –i.e. 54% of *S0's* deaths are avoided-, 1.3 million in *TZ* and 598,000 in *CM*), followed by the Universal Access (*S2a*) scenario (187,000 in *SZ*, 1 million in *TZ* and 441,000 in *CM*). The “Aid Freeze” scenario would only avoid 27,000 deaths in *SZ* (respectively 155,000 & 52,000 in *TZ* & *CM*) compared with *S0*.

**Table 2:** Epidemiological Impacts of 4 ART Coverage Scenarios in 3 Countries

Epidemiology			Late 2000's	Mid 2030's			
**Country**	**Indicators**		Initial Characteristics	Scenario *S0* No Access	Scenario *S1* Aid Freeze	Scenario *S2a* Universal Access	Scenario *S2b* Extended Universal Access
**Swaziland**	**Prevalence**	HIV Prevalence *(in percent)*	25.88	21.91	22.45	26.81	12.86
2007 – 2037		PLWHIV *(in thousands)*	151	350	362	458	223
		Proportion of HIV++ among PLWHIV	0.310	0.290	0.312	0.459	0.462
		Number of Adults aged 15-49 *(in thousands)*	582	1,600	1,611	1,708	1,737
	**Deaths**	Deaths from 2032 onwards *(in thousands)*	n/a	111	108	67	52
		Deaths from 2032 onwards due to HIV *(in percent)*	n/a	79.9	79.3	67.4	29.6
		Cum. Deaths from 2007 onwards *(in thousands)*	n/a	439	412	252	201
	**Infections**	Cum. Infections from 2007 onwards *(in thousands)*	n/a	615	615	615	272
**Tanzania**	**Prevalence**	HIV Prevalence *(in percent)*	6.35	5.35	5.47	6.68	3.38
2009 – 2034		PLWHIV *(in thousands)*	1,195	1,972	2,021	2,500	1,270
		Proportion of HIV++ among PLWHIV	0.32	0.26	0.28	0.41	0.45
		Number of Adults aged 15-49 *(in thousands)*	18,828	36,894	36,952	37,428	37,612
	**Deaths**	Deaths from 2029 onwards *(in thousands)*	n/a	643	617	441	345
		Deaths from 2029 onwards due to HIV *(in percent)*	n/a	57.2	56.5	37.4	16
		Cum. Deaths from 2009 onwards *(in thousands)*	n/a	3,144	2,989	2,135	1,811
	**Infections**	Cum. Infections from 2009 onwards *(in thousands)*	n/a	3,655	3,655	3,655	1,811
**Cameroon**	**Prevalence**	HIV Prevalence *(in percent)*	5.13	4.67	4.78	6	2.90
2009 – 2034		PLWHIV *(in thousands)*	471	863	884	1,124	545
		Proportion of HIV++ among PLWHIV	0.3	0.27	0.29	0.44	0.48
		Number of Adults aged 15–49 *(in thousands)*	9,169	18,480	18,504	18,733	18,825
	**Deaths**	Deaths from 2029 onwards *(in thousands)*	n/a	413	404	311	264
		Deaths from 2029 onwards due to HIV *(in percent)*	n/a	45	43.9	26.3	9.7
		Cum. Deaths from 2009 onwards *(in thousands)*	n/a	1,817	1,765	1,376	1,219
	**Infections**	Cum. Infections from 2009 onwards *(in thousands)*	n/a	1,584	1,584	1,584	760

**Figure 3:**
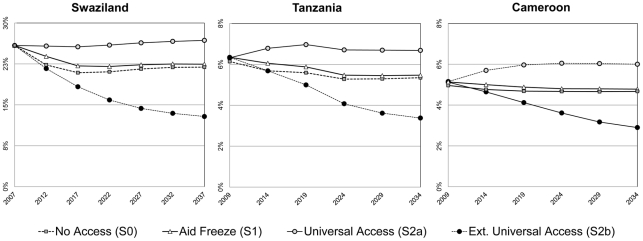
HIV prevalence rates. Evolution of HIV prevalence rates overtime in the three studied countries as per the four hypothetical ART access scenarios.

Because of the high mortality associated with HIV in the absence of ART, a decrease in HIV prevalence rates, shown in Figure 3, can be predicted for the “No Access” Scenario (*S0*) in the three examined countries (by 4% in *SZ*, 1% in *TZ* and 0·5% in *CM*). Treating individuals with ART produces a mechanical increase of HIV prevalence: HIV-infected individuals live longer, which results in a higher number of HIV carriers than in the scenario *S0*: the prevalence rate of *S0* is slightly lower than in *S1* but considerably lower than in *S2a* (Figure 3). In the Extended Universal Access Scenario (*S2b*), the differential in prevalence rates due to the surviving HIV-infected population (these survival rates are even higher in *S2b* than in *S2a*) is reversed by the reduction of incidence: 343,000 new infections can be avoided over 30 years in *SZ*, 1.8 million in *TZ* and 824,000 in *CM* over 25 years (due to the preventive effect of the program), such that HIV prevalence is reduced by half at the end of the micro-simulation process (it diminishes by 13 % in *SZ*, 3% in *TZ* and 2% in *CM*). Since mortality and infection rates of HIV- individuals are equal in *S0*, *S1* and *S2a*, the cumulated numbers of new infections observed over the simulation process are, by construction, equal across these options.

### Economic Impact

The annual costs associated with the “Aid Freeze” scenario remain stable in the three countries as the number of treated individuals remains constant (Table 3). For the Universal Access scenario (*S2a*), 753 million USD would be disbursed in *SZ* to cover 210,000 individuals for the final period 2032–2037 (the annual costs of the program are multiplied by 5 over 30 years), while the expenditures reach respectively 3.7 and 1.8 billion USD in *TZ* and *CM* for the final period (respectively three and four times the first period's costs). The overall spending involved by the Extended Universal Access scenario *S2b* are up to 15% higher than the previous figures. However the time-structure of these costs differs in this latter scenario: annual costs are important in the first period of the simulation (the program initiates ART for an increased portion of the HIV+ population) but increase more slowly afterwards (they only double in the three countries) as the preventive effect of earlier treatment implies that new HIV infections are diminished.

Due to the general growth of the economy (resulting from demographic & epidemiological changes solely), an increase in the GDP per capita occurs in the “No Access” Scenario (*S0*) at the end of the micro-simulation process (by 9·8% in *SZ*, 0·7% in *TZ* and 4·7% in *CM*), but GDP growth-rates are higher when ART programs are initiated: a surplus, positively correlated with the coverage rate of the population can be generated through increased productivity of treated HIV-infected workers.

**Figure 4:**
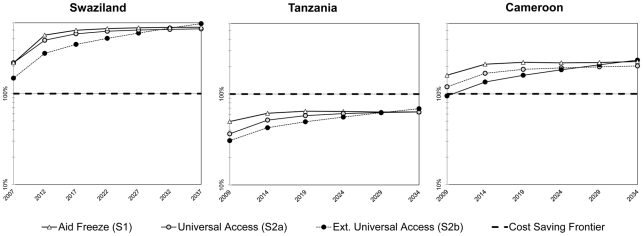
Self Financing Ratios. Time trends in the cost-benefit measures of ART programs in the three examined countries. The Y axis shows, on a logarithm 10 scale, the ratio between the cumulated GDP gains (compared with the No Access scenario *S0*) and the ART program cumulated costs.

To what extent ART programs may be cost-saving when such positive macroeconomic impact is taken into account? The answer depends on the countries' GDP levels (as ARVs prices were assumed constant across the three countries of our modeling exercise). For both Swaziland and Cameroon, when compared to the counterfactual benchmark (no access to ART at all), all scenarios of ART coverage appear to be cost saving: the production gains they allow are higher than their cumulated costs (as shown in Figure 4, the self-financing ratios exceed 100% in *CM* and *SZ*). In both countries, the aid freeze scenario (*S1*) is the most cost saving at the beginning of the simulation but after 2030, the extended universal access scenario (*S2b*) provides the maximal net benefit and dominates (in terms of SFR) the other scenarios in the long run (Figure 4 and Table 3). In Tanzania, the extended universal access scenario (*S2b*) also dominates over the long run. However, since the GDP per capita is far lower than in Cameroon and Swaziland, GDP gains compensate only partially the total costs of ART in the three scenarios: 69% for *S2b*, and respectively 64% and 63% for *S1* and *S2a*. Financing ART programs in *TZ* clearly implies a net cost for the economy: only 69% of total costs are covered by GDP gains in 2034 in *S2b*. However, in all scenarios, the net-cost per life year saved is smaller than 1000$, even when using the very conservative assumption that each life saved only represents on average five years of additional life expectancy.

**Table 3:** Economic Impacts of 4 ART Coverage Scenarios in 3 Countries.

Economics			Late 2000's	Mid 2030's			
**Country**	**Indicator**		Initial Characteristics	Scenario *S0* No Access	Scenario *S1* Aid Freeze	Scenario *S2a* Universal Access	Scenario *S2b* Extended Universal Access
**Swaziland** 2007 –2037	**ART Coverage**	ART Coverage of HIV++ population *(in percent)*	42	0	17	100	149
		Individuals under ART *(in thousands)*	20	0	20	210	154
	**ART Costs**	Total ART Cost from 2032 onwards *(in millions USD)*	n/a	0	72	753	590
		Total ART Cost from 2007 onwards *(in millions USD)*	n/a	0	496	3,115	3,336
	**GDP**	GDP per Capita *(in USD)*	4,466	4,905	4,921	5,068	5,115
		Overall GDP *(in billions USD)*	2.6	7.85	7.93	8.66	8.88
		GDP gap from 2007 onwards *(in millions USD)*	n/a	Reference	2,668	16,067	19,630
		GDP gains / ART Costs from 2007 onwards	n/a	Reference	5.37	5.16	5.88
**Tanzania** 2009 – 2034	**ART Coverage**	ART Coverage of HIV++ population *(in percent)*	31	0	19	100	149
		Individuals under ART *(in thousands)*	119	0	110	1,103	856
	**ART Costs**	Total ART Cost from 2029 onwards *(in millions USD)*	n/a	0	402	3,670	3,320
		Total ART Cost from 2009 onwards *(in millions USD)*	n/a	0	2,672	15,980	18,432
	**GDP**	GDP per Capita *(in USD)*	699.5	704.2	704.5	707.2	708.3
		Overall GDP *(in billions USD)*	13.17	25.98	26.03	26.47	26.64
		GDP gap from 2009 onwards *(in millions USD)*	n/a	Reference	1,696	10,138	12,712
		GDP gains / ART Costs from 2009 onwards	n/a	Reference	0.64	0.63	0.69
**Cameroon** 2009 – 2034	**ART Coverage**	ART Coverage of HIV++ population *(in percent)*	25	0	14	100	149
		Individuals under ART *(in thousands)*	35	0	36	492	390
	**ART Costs**	Total ART Cost from 2029 onwards *(in millions USD)*	n/a	0	131	1,769	1,516
		Total ART Cost from 2009 onwards *(in millions USD)*	n/a	0	848	7,239	8,060
	**GDP**	GDP per Capita *(in USD)*	2,062	2,159	2,160	2,171	2,176
		Overall GDP *(in billions USD)*	18.9	39.9	39.96	40.67	40.95
		GDP gap from 2009 onwards *(in millions USD)*	n/a	Reference	1,911	14,989	18,973
		GDP gains / ART Costs from 2009 onwards	n/a	Reference	2.27	2.07	2.35

The long term dominance of the Extended Universal Access Scenario on the others requires further examination of the model's hypotheses. Indeed, this scenario has, compared to the others, three relative advantages: the spread of the epidemic, the progression pace to the symptomatic stage of HIV and the mortality rate under treatment are all decreased. These three modifications have notable effects on the number of live saved and the self financing ratio (through program costs and/or GDP gains). We propose here eight sensitivity tests, wherein each key parameter from the microsimulation is altered. Table 4 provides estimates of both SFR and lives saved -compared to *S0-* at the end of the simulation process for the core analysis described previously and the alternative stipulations in the three studied countries. Sensitivity analyses V1-V3 weaken (separately) each relative advantage of *S2b*. In V1, ϕ, the strength of the prevention effect, is lowered from 0.7 to 0.5. *S2b* becomes less profitable than *S1* (and sometimes *S2a*). When ψ (restraining the progression to HIV++) is decreased from 0.3 to 0.1 in V2, *S2b* becomes dominated by *S1* except in *SZ* but remains more profitable than *S2a*. The same conclusion can be drawn when the mortality under ART when initiated early, u^*^, is increased from 0.1 to 0.15. We then symmetrically assume that the mortality rates under treatment (late initiation) are augmented from 0.2 to 0.3: *S1* and *S2a* are even less efficient than in the core analysis (V4). If we assume that a preventive effect exists for *S2a* (raising the parameter from zero to 0·2), *S2a* slightly dominates *S2b* (and thus *S1*) in the long run (V5). Thanks to the preventative effect of treatment, *S2b* saves additional lives and becomes more efficient when the mortality rate of the asymptomatic HIV population is increased (v parameter, V6). We finally conduct two robustness tests on the microeconomic components of the model that conserve policy rankings (a decrease of presence rates under ART, V7, and a decrease in the price of 2^nd^ line HIV medicines). These eight sensitivity analyses demonstrate two things: the financial sustainability of ART programs is robust to any change, and seems to depend more on the initial GDP-per-capita, rather than on the parameter used for the transition-matrix (programs are always cost-effective in Cameroon and Swaziland, but not in Tanzania); the precise ranking of S1, S2a and S2b can change depending on parameter values, but the general sketch is that S2b tends to dominate the alternatives in the long run in terms of SFR and is anyway the most life-saving option throughout the simulation.

**Table 4:** Sensitivity Analyses.

Last Period Outcomes		Main Analysis		V1		V2		V3		V4		V5		V6		V7		V8	
				Φ(*S2b*) from 0.7 to 0.5		Ψ(*S2b*) from 0.3 to 0.1		u (S*2b*) from 0.1 to 0.15		u (*S1 & S2a*) from 0.2 to 0.3		Φ (*S2a*) from 0 to 0.2		v from 0.005 to 0.05		Presence of patients from 0.95 to 0.85		2^nd^ line costs from $600 to $400	
		*SFR*	*LS*	*SFR*	*LS*	*SFR*	*LS*	*SFR*	*LS*	*SFR*	*LS*	*SFR*	*LS*	*SFR*	*LS*	*SFR*	*LS*	*SFR*	*LS*
**SZ 2037**	*S1*	5.37	27	5.37	27	5.37	27	5.37	27	**5.16**	**24**	5.37	27	5.37	27	4.52	27	6.54	27
	*S2a*	5.16	187	5.16	187	5.16	187	5.16	187	**4.90**	**148**	**5.92**	**196**	5.16	187	4.25	187	5.98	187
	*S2b*	5.88	238	**5.02**	**233**	**5.56**	**236**	**5.53**	**221**	5.88	238	5.88	238	**6.38**	**250**	5.30	238	7.04	238
**CM 2034**	*S1*	2.27	52	2.27	52	2.27	52	2.27	52	**2.15**	**45**	2.27	52	2.27	52	1.87	52	2.82	52
	*S2a*	2.07	441	2.07	441	2.07	441	2.07	441	**2.02**	**361**	**2.37**	**468**	2.07	441	1.71	441	2.42	446
	*S2b*	2.35	598	**2.10**	**584**	**2.18**	**591**	2.21	490	2.35	598	2.35	598	**2.51**	**609**	2.10	598	2.87	598
**TZ 2034**	*S1*	0.64	155	0.64	155	0.64	155	0.64	155	**0.60**	**134**	0.64	155	0.64	155	0.52	155	0.76	155
	*S2a*	0.63	1010	0.63	1010	0.63	1010	0.63	1010	**0.60**	**800**	**0.70**	**1041**	0.63	1010	0.52	1010	0.72	1010
	*S2b*	0.69	1334	**0.62**	**1318**	**0.63**	**1315**	**0.65**	**1243**	0.69	1334	0.69	1334	**0.73**	**1388**	0.61	1334	0.82	1334

## Discussion

The use of micro-simulation techniques combined with the availability of detailed data on economic behaviors at individual levels in three African countries allowed us to forecast the impact of alternative scenarios for access to ART throughout the whole economy. Previous economic literature on the cost-effectiveness of ART programs has mainly focused on the microeconomic consequences of alternative use of resources devoted to the fight against the HIV pandemic: trade-off between prevention and treatment or trade-off between alternative threshold criteria for initiating ART or switching to second line regimens [37]–[41]. Although such approaches are very useful to help optimize treatment and comprehensive strategies and adapt them to low-resource settings [42], they ignore *de facto* the macroeconomic benefits induced by procuring treatment to the labor force. Our micro-simulation based modeling improves the contribution of economic evaluations to policy making decisions by adopting a concept of “net total costs and benefit” integrating GDP-gains, as in Resch et al. [43].

Not surprisingly, the human toll that would be paid by freezing ART coverage at the current levels, rather than pursuing the way forward the UNGASS goal of universal access, would be quite enormous. In such freezing scenario, the situation in terms of future AIDS-related deaths would not be very different in the 2030's from the counterfactual scenario of no-access at all to ART (by comparison, no more than 6% of additional deaths could be avoided by the current level of coverage). In contrast, universal access programs could save six times more lives. Our results also show that although universal access scenarios are logically more costly in the short term they are indeed the more cost-beneficial in the long term, when taking into account their macroeconomic consequences. The evaluations performed for Swaziland and Cameroon show that Universal Access scenarios would imply net cost-savings at the scale of the society.

The growth rate of an economy and future national wealth depend on the choices made (i.e. the people saved have a productive value which adds to the social value of human lives). The extra economic value created provides funding for treatment access programs such that Universal Access is found to be the most economically feasible option. In purely economic terms, Universal Access may be considered as an investment in productive human capital, with a positive 'Keynesian-flavored' multiplier (public spending with higher returns than initial expenditures). Reciprocally, scenarios dealing with freezing programs (e.g. reduced international aid because of the world financial crisis) despite (initially) yielding higher tax returns generate smaller benefits in the long term and have recessive effects on the economy.

Our results convey two additional messages to ongoing international debates. First, they tend to confirm [44] that in spite of the mechanical increase of direct treatment costs, the extended coverage implied by the recent revision of WHO guidelines, wherein ART is initiated at higher levels of CD4 cell counts (350 cc/mm^3^), is cost-effective when including its whole macroeconomic impact. Second, they confirm that the macroeconomic consequences of universal access to ART are highly sensitive to its potential impact on HIV transmission and consequently on the trajectory of the HIV epidemic globally [45]. This brings an additional argument for the urgent need of large scale randomized experiments to provide definitive data about the impact on the epidemic of the so-called “test and treat” or “treatment as prevention” strategy [46]. Future research could also investigate whether “test and treat” strategies could be economically sustainable. As results of the present paper suggest, the answer would notably depend on the ability of such a strategy to reduce inter-individual transmission, patients' mortality and control viral load.

Of course, there are two obvious limitations when extrapolating our results to the current situation of HIV funding. Our estimations are limited to only three countries and may not be valid for other African contexts. However, the three selected countries are quite representative of the heterogeneity of the epidemiological and economic situations in sub-Saharan Africa. Moreover, the reality of HIV funding in the next future may be less dramatic than the extreme “freezing” scenario which we selected. Despite the financial crisis, OECD figures showed continuing progress in total net official development assistance (ODA) in both 2008 and 2009 [47]. Significant efficiency gains in the use of already available funding may be obtained through improved synergies with other Millennium Development Goals-related programs and health systems strengthening [48] and through the use of innovations in care delivery to promote broader healthcare reforms [49]. However, recent trends, such as the limited 20% increase in donor pledges for the next three years for the GFATM, show that the threat of a significant delay in the scaling up of universal access to ART is a very real one.

In addition, a number of methodological limitations should be acknowledged. For example, individual's behaviors included in our modeling are quite static. We do not consider questions of adherence or therapeutic failure in an in-depth manner: a “delivered” treatment in our model is one which operates with a given percentage of efficacy (without consideration for age, illness duration or treatment duration) and the probabilities of survival of cohorts of agents benefitting from ART are estimated on the basis of published results in the literature. Another limitation is that our “net cost” approach does not really tackle the programs' feasibility by countries' healthcare systems [50]: if a nation experiences a shortage in the health workforce, Universal Access may be unattainable despite being budgeted and economically sustainable. It could therefore be quite interesting to examine whether the large procurement of treatment to the population, besides generating GDP gains, could enhance the public-health sector. It can also be noticed that we do not take into account the “value” of human life or the quality of added surviving years. A large improvement on the current analysis could be provided by incorporating these dimensions, mainly for countries in which the “net cost” remains positive (such as Tanzania). Concerning this latter point, the model used for this analysis has the strong advantage of being applicable to any country covered by the DHS program (26 surveys were conducted, including 20 in Sub-Saharan Africa). There is, then, the possibility to create a list of countries in which programs are unambiguously cost saving, and (symmetrically) a list of countries in which we must add an ethical “value of human life” argument in order to advocate in favor of universal ART access.

In spite of these limitations, the general message underlined by our results is that scaling-up strategies for universal access to ART, which are more costly in the short term, remain the best economic choice in the long term. Renouncing or significantly delaying the achievement of this goal, due to “legitimate” short term budgetary constraints would be a misguided choice.

## Supporting Information

Appendix S1 **Detailed methodology of the Micro-simulation Model.**
(DOCX)

Figure S1 (TIFF)
