# Supplementary material for: The Macroeconomic Consequences of Renouncing to Universal Access to Antiretroviral Treatment for HIV in Africa: A Micro-Simulation Model
Source: PLoS One. 2012 Apr 13;7(4):e34101. doi: 10.1371/journal.pone.0034101 (PMC3325986; doi:10.1371/journal.pone.0034101)

**Appendix: Detailed methodology of the Micro-simulation Model**

**Categorization of PLWHIV in two stages**

HIV Blood tests from DHS surveys provide information on whether or not an individual is infected with HIV, regardless the severity of HIV (that can be measured by e.g. CD4 count). As shown in Table S1 (columns 3-4), HIV prevalence is a concave function of age, women being infected by HIV at a younger age than men, such that the maximal prevalence rates are experienced by female aged 25-35 and by 35-45 men. In resource-poor communities, medical facilities are sometimes poorly equipped, and it is not possible to use CD4 and viral load test results to determine the right time to begin antiretroviral treatment.

The WHO has therefore developed a staging system for HIV disease based on clinical symptoms, which may be used to guide medical decision making. In 2006, the WHO recommended to initiate ART for HIV infected people with a CD4 cell count ≤200 cells/µl, those in clinical stage III with a CD4 cell count ≤350 cells/µl and those with a diagnosis of WHO stage IV disease [[25](#_ENREF_25)]. We use this definition of treatment need for categorizing the HIV-population into two groups: those in the symptomatic HIV infection stage (denoted as HIV++) who require ARVs according to the WHO 2006 guidelines and those in the clinically asymptomatic stage (HIV+) that should not receive ARVs according to these guidelines.

To do so, we randomly incorporated the HIV-positive individuals into one of the two groups using the estimated proportion of individuals needing ART among PLWHIV estimated by WHO/UNAIDS [[5](#_ENREF_50)1]. There is some evidence that this latter proportion $\tau$is higher among the oldest HIV positive individuals. This ratio $\tau$ was therefore heterogenized across genders $Z=\left( 1;2 \right)$ and 5-years-age-groups $X=\left( 1;\cdots;7 \right)$ using a geometric sequence $\tau_{X;Z}$ with common ratio$\left( 1+\alpha\right)$, where $\alpha=0.1$ :

$$\tau_{X+1;Z}=\left( 1+\alpha\right)\cdot\tau_{X;Z} ;\text{suc}\text{h}\text{ t}\text{h}\text{at}\text{ }\frac{1}{\hat{N}_{Z;PLWHIV}}\sum_{X} \left[ \hat{N}_{X;Z;PLWHIV}\cdot\tau_{X;Z} \right]=\tau$$

Where $\hat{N}_{X;Z;PLWHIV}$is the age-gender specific number of PLWHIV in the sample (regardless the stage of HIV). In the model, we fixed the level of $\alpha$ at 10%, which results in the health-status distributions presented in Table S1, columns 7-8. With $\tau$ estimated by WHO/UNAIDS at around 30% in the three countries (Table 1), the calculated age-gender specific ratios $\tau_{X;Z}$range from 22% to 43% (Table S1).

**Computation of Transition Probabilities**

***Probabilities of dying: Computation of*** $\boldsymbol{P}_{\boldsymbol{03}}\boldsymbol{,}\boldsymbol{P}_{\boldsymbol{13}}\boldsymbol{,}\boldsymbol{P}_{\boldsymbol{23}}\boldsymbol{\&} \boldsymbol{P}_{\boldsymbol{22}}$

Demographic and Health Surveys include a sibling survivorship history. This module provides a detailed account of the survivorship of all of the live-born children of the respondent’s mother: for living siblings, current age was collected; for deceased siblings, age at death and years since death were collected. Age and gender specific mortality rates are obtained by dividing the age-gender specific number of deaths $D_{X;Z}$ occurring in the 0-6 years preceding the interview by the number of person-years of exposure to death$E_{X;Z}$. However, due to the small number of deaths occurring in several age-groups, the estimated rates are subject to considerable sampling variation. These rates where then transformed into age-gender specific probabilities of dying from any cause between ages $X$ and$X+1$: $P_{*3}^{X;Z}=\left[ 1-\left( 1-{D_{X;Z}}/{E_{X;Z}} \right)^{5} \right]$ (refer to Table S1, columns 5-6, for values). These probabilities were then decomposed into the three causes of mortality within the model’s sub-populations:

$$P_{*3}^{X;Z}= \frac{1}{\hat{N}_{X;Z}}\left[ \hat{N}_{X;Z;HIV-}\cdot P_{03}^{X;Z}+\hat{N}_{X;Z;HIV+}\cdot P_{13}^{X;Z}+\hat{N}_{X;Z;HIV++}\cdot P_{23}^{X;Z} \right] (a)$$

Several works from the literature on the survival of HIV positive individuals enable us to write $P_{13}^{X;Z}$ and $P_{23}^{X;Z}$ as functions of$P_{03}^{X;Z}$. Therefore, $P_{03}^{X;Z}$ could then be computed from $P_{*3}^{X;Z}$.

It can be assumed that HIV positive individuals in need of treatment die in the subsequent five years if they do not receive ARVs: reviews on the time from ART eligibility to death by AIDS indicate a median time of about 3 years for those without treatment [[52](#_ENREF_51),[53](#_ENREF_52)].

ART strongly enhances the survival rate of the HIV++ individuals. In the context of the 2006 eligibility for treatment criteria, Stover et al. (2008, [[5](#_ENREF_52)3]) undertook a literature review showing a median 24-month survival rate on ART of 84%, while Etard (2006, [[5](#_ENREF_53)4]) and Leger (2009, [[5](#_ENREF_54)5]) indicate a survival rate of 75% at 5 years after diagnosis. Furthermore, the early initiation of ART strongly reduces mortality among patients [[56-5](#_ENREF_55)8]. Therefore, we postulate that mortality rates are substantially reduced with ART, but remain greater than those of the HIV- population: we inflate $P_{03}^{X;Z}$ by a constant$u$. We first set $u$ at 0.2 but this parameter can be lowered with more effective treatments due to earlier initiation $\left( u=0.1 \right)$or medical progress. We also tested in sensitivity analyses values of $u=0.3$ instead of 20% (and $u=$0.15 instead of 0.1 in the case of early access).

We can then define $P_{23}^{X;Z}$ in (a): $P_{23}^{X;Z}=\left\{ \begin{matrix} 1 & \text{without ART} \\ P_{03}^{X;Z}+u & \text{otherwise} \end{matrix} \right.$(see Table S1, columns 17-18)

Symmetrically, given that the probabilities $P_{20}^{X;Z}$ and $P_{21}^{X;Z}$ are null and that $P_{2*}^{X;Z}=1$, we have

$P_{22}^{X;Z}=\left( 1-P_{23}^{X;Z} \right)$.

The value of $P_{23}^{X;Z}$ depends explicitly on whether HIV++ individuals were likely to be under ART. $\mathrm{TC}_{T_{0-}}$, the proportion of individuals in need of treatment (according to 2006 eligibility criteria) receiving ART in the 0-6 years preceding the interviews had therefore to be estimated. Without sufficient retrospective data, the country-specific proportions $\mathrm{TC}_{T_{0-}}$ are approximated by the ART coverage rates estimated at the same year of DHS interviews. These figures are 0.71% in Tanzania for 2004, 8.75% in Cameroon for 2004 and at 42.37% in Swaziland for 2008 [[51](#_ENREF_50)].

We assume that individuals in the asymptomatic stage of HIV are “slightly” more likely to die in the subsequent period than uninfected individuals as the former may have / have had more risky behaviors. $P_{03}^{X;Z}$ is thus (solely) inflated by a constant $v=0.005$ (alternatively set at 0.05 for sensitivity tests) such that we can rewrite $P_{13}^{X;Z}$ in (a) by:

$P_{13}^{X;Z}= P_{03}^{X;Z}+v$ (see Table S1 columns 15-16)

$P_{03}^{X;Z}$ can be written from (a) as a function of the overall death probabilities $P_{*3}^{X;Z}$ , of the parameters $u$ (corresponding to late initiation) and $v$ and of the retrospective ART coverage rates $\mathrm{TC}_{T_{0-}}$:

$$P_{03}^{X;Z}=\frac{\hat{N}_{X;Z}\cdot P_{*3}^{X;Z}-\hat{N}_{X;Z;HIV+}\cdot v-\hat{N}_{X;Z;HIV++}\cdot\mathrm{TC}_{T_{0-}} \cdot u-\hat{N}_{X;Z;HIV++}\cdot\left( 1-\mathrm{TC}_{T_{0-}} \right)}{\hat{N}_{X;Z;HIV-}+\hat{N}_{X;Z;HIV+}+\hat{N}_{X;Z;HIV++}\cdot\mathrm{TC}_{T_{0-}}}$$

This decomposition results in the computation of gender and health-status specific probabilities of dying between ages $X$ and$X+1$, which are presented in Table S1. Four probabilities, associated with Swahili females aged 20-24 or 30-34, take negative and thus unrealistic values: these values are hereafter censored to zero. Several reasons can be advanced: underestimated$P_{*3}^{X;Z}$, inappropriate distribution of $\tau_{X;Z}$ (logistic rather than linear, incorrect$\mathrm{TC}_{T_{0-}}$).

***Seroconversion: Computation of*** $\boldsymbol{P}_{\boldsymbol{01}}$ ***and*** $\boldsymbol{P}_{\boldsymbol{00}}$

Agents are connected each other by their risk of seroconversion. With $P_{01}^{i}$the probability of agent $i$ to experience a transition from HIV- to HIV+, we state that$P_{01}^{i}=f(E_{1}, \ldots E_{j},\ldots) \forall j\neq i$: as for all infectious diseases, the individual risk of infection is endogenously determined by the health status of the other agents in the population$\left( \boldsymbol{E}_{\boldsymbol{1}}, \ldots\boldsymbol{E}_{\boldsymbol{j}}\ldots\right)$.

For our purpose we then propose to build contamination rates using the following assumption:

$$P_{01}^{X;Z}=\left( 1-\Phi\right)\cdot\gamma\cdot\frac{\hat{N}_{X+1;Z;HIV+}}{\hat{N}_{X+1;Z}}$$

In this last formulation, the probability that an agent of age-group $X$ becomes infected by HIV within the subsequent five years linearly depends on the prevalence of HIV among the next age-group cohort observed in DHS. This prevalence rate is multiplied by two parameters comprised between zero and one: $\left( 1-\Phi\right)$ and$\gamma$. The factor $\gamma$ can capture (and vary with) behavioral considerations: sexual partnership patterns, use of condoms, etc. We use $\gamma$ to reproduce the current patterns of HIV infection at national levels. In the absence of longitudinal population-based surveys, $\gamma$ is calibrated with the number of new infections estimated by WHO/UNAIDS for the five years following DHS interviews [[51](#_ENREF_50)]. $\gamma$ has been estimated at 0.518 in Swaziland, at 0.388 in Tanzania and at 0.511 in Cameroon, resulting in the age-gender specific infection rates provided in Table S1, columns 9-10. For purpose of simplification, we assume $\gamma$ constant over time. Note that any variation of this external data would ceteris paribus affect all scenarios’ outcomes similarly.

$\Phi$is included in function $f$ to take into account the impact of public policies which may counter the dynamics of the epidemic. For example, high antiretroviral coverage with early initiation can generate a preventive effect on the spread of HIV infections [[28](#_ENREF_28)]. $\Phi$has been calibrated with the goal to replicate reasonable forecasts of the shifts in the epidemics due to this effect. We set$\Phi$at 0.7: at the end of the simulation process the incidence rate is reduced by around 60% compared to *S1*, which is far too low to eliminate the HIV epidemic advocated by Granich and his colleagues. An alternative value of 0.5 was used for sensitivity analysis.

The probability to remain HIV- in the subsequent five years is given by$P_{00}^{X;Z}=\left( 1-P_{01}^{X;Z}-P_{03}^{X;Z} \right)$.

***Progression to Advanced HIV***

An analysis of pooled data from eight different studies indicates that the median time from infection to requiring treatment at 2006 guidelines of about 8 years [[5](#_ENREF_58)9]. The speed of progression from infection to advanced HIV varies with age and gender, with older people advancing more rapidly and women tending to become infected at younger ages than men. The probability of an individual aged in a range $X$ to progress from HIV+ to HIV++ in the subsequent five years is defined by the product between $\beta$, a calibration parameter, $\psi$, a scalar that diminishes the transition rate $P_{12}^{X;Z}$ in case of early access to ART [60], and $\tau_{X+1;Z}$, the proportion of advanced HIV individuals among PLWHIV from the subsequent age cohort $\left( X+1 \right)$:

$$P_{12}^{X;Z}=\psi\cdot{\beta\cdot\tau}_{X+1;Z}$$

The value of $\beta$ is calibrated in order to fit our projections with the number of individuals in need of treatment forecasted by UNAIDS[[5](#_ENREF_50)1].$\beta$ was estimated at 1.3 in Swaziland and Cameroon and at 1.27 in Tanzania, resulting in the age-gender specific probabilities provided in Table S1, columns 13-14. (We also checked that our transition probabilities confirm epidemiological studies [57]). $\psi$ is assumed to be equal to 70% for HIV+ individuals benefiting from early ARV procurement in scenario *S2b* and equal to 100% otherwise (i.e. it has no impact). We alternatively use a value of 90% for $\psi$ for sensitivity tests.

The probability to remain HIV+ in the subsequent five years is given by$P_{11}^{X;Z}=\left( 1-P_{12}^{X;Z}-P_{13}^{X;Z} \right)$.

**Testing the model’s validity: an external validation**

In order to test the reliability of the Markovian model, it is essential to check the accuracy of the transition rates estimated. We thus compare the predictions from the model obtained in the Aid Freeze Scenario (this scenario is the closest from reality) with real data (Figure S1). Two key estimates are generally used to examine the course of the HIV epidemic: the HIV prevalence and incidence rates (estimated by UNAIDS [[51](#_ENREF_50)]).

In Cameroon, the simulated prevalence rates match UNAIDS estimates in 2004 (5.44% against 5.40% for UNAIDS) and 2009 (5.13% against 5.30%, but the ART coverage rate is lower with our modeling). The THIS 2003-04 slightly overestimate UNAIDS data in 2004 and 2009 but present a similar decrease in HIV prevalence. Turning to Swaziland, prevalence data is not available for 2013 and cannot be compared with our prediction; however the SDHS 2007-08 matches UNAIDS data (Figure S1).

To be compared with forecasts of the microsimulation model, the annual incidence rate calculated by UNAIDS was transformed into a 5-years incidence rate as follows:

$$I\left( t;t+5 \right)=1-\prod_{i=0}^{i=4} 1-I\left( t+i \right);$$

Where $I\left( t+i \right)$ is the annual incidence rate estimated at $\left( t+i \right)$. The 5-years incidence rates (between 2004 and 2009) in Cameroon and Tanzania fit UNAIDS data (2.71% vs. 2.72% in *TZ*; 2.74% in *CM* vs. 2.78%; Figure S1). In Swaziland, UNAIDS estimates of HIV incidence in the 2008-13 period have not been published; we observe that our microsimulation forecast matches the trend-projection of 2003-08 UNAIDS data as well as Spectrum incidence forecasts for the 2008-13 period [61] .

**References**

51. UNAIDS (2010) AIDS Info. Available From: <http://www.aidsinfoonline.org/>. Accessed 2012 Mar 2.

52. Zwahlen M., Egger M. (2006) Progression and mortality of untreated HIV-positive individuals living in resource-limited settings: Update of Literature review and evidence synthesis.

53. Stover J, Johnson P, Zaba B, Zwahlen M, Dabis F, et al. (2008) The Spectrum projection package: improvements in estimating mortality, ART needs, PMTCT impact and uncertainty bounds. Sex Transm Infect 84 Suppl 1: i24-i30.

54. Etard JF, Ndiaye I, Thierry-Mieg M, Gueye NF, Gueye PM, et al. (2006) Mortality and causes of death in adults receiving highly active antiretroviral therapy in Senegal: a 7-year cohort study. AIDS 20: 1181-1189.

55. Leger P, Charles M, Severe P, Riviere C, Pape JW, et al. (2009) 5-year survival of patients with AIDS receiving antiretroviral therapy in Haiti. N Engl J Med 361: 828-829.

56. Siegfried N, Uthman OA, Rutherford GW (2010) Optimal time for initiation of antiretroviral therapy in asymptomatic, HIV-infected, treatment-naive adults. Cochrane Database Syst Rev: CD008272.

57. Ford N, Kranzer K, Hilderbrand K, Jouquet G, Goemaere E, et al. (2010) Early initiation of antiretroviral therapy and associated reduction in mortality, morbidity and defaulting in a nurse-managed, community cohort in Lesotho. AIDS 24: 2645-2650.

58. Kitahata MM, Gange SJ, Abraham AG, Merriman B, Saag MS, et al. (2009) Effect of early versus deferred antiretroviral therapy for HIV on survival. N Engl J Med 360: 1815-1826.

59. Todd J, Glynn JR, Marston M, Lutalo T, Biraro S, et al. (2007) Time from HIV seroconversion to death: a collaborative analysis of eight studies in six low and middle-income countries before highly active antiretroviral therapy. AIDS 21 Suppl 6: S55-63.

60. Sterne JA, May M, Costagliola D, de Wolf F, Phillips AN, et al. (2009) Timing of initiation of antiretroviral therapy in AIDS-free HIV-1-infected patients: a collaborative analysis of 18 HIV cohort studies. Lancet 373: 1352-1363.

61. Futures Institute (2011) Spectrum 4.39 Available from: <http://www.futuresinstitute.org/Pages/Spectrum.aspx>. Accessed 2012 Mar 2.

| **Table S1 : Transition Probabilities by Age & Gender** | | | | | | | | | | | | | | | | | |
| --- | --- | --- | --- | --- | --- | --- | --- | --- | --- | --- | --- | --- | --- | --- | --- | --- | --- |
| **Country** | Age | **Exogenous Data** | | | | **Computed Rates** | | | | | | | | | | | |
|  |  | **HIV Prevalence rate** | | **P(dying) between X and X+1** | | $\boldsymbol{\tau}$  **(ART Need)** | | **P_01_**  **(HIV- to HIV+)** | | **P_03_**  **(HIV- to Death)** | | **P_12_**  **(HIV+ to HIV++)** | | **P_13_**  **(HIV+ to Death)** | | **P_23_**  **(ART to Death)** | |
|  |  | M | F | M | F | M | F | M | F | M | F | M | F | M | F | M | F |
| Swaziland | 15-19 | 1.87% | 10.07% | 1.10% | 2.03% | 22.76% | 24.32% | 4.79% | 14.57% | 0.81% | 0.38% | 32.55% | 34.78% | 1.31% | 0.88% | 20.81% | 20.38% |
|  | 20-24 | 12.34% | 38.41% | 2.82% | 5.81% | 25.04% | 26.75% | 10.42% | 17.99% | 0.74% | 0%* | 35.80% | 38.25% | 1.24% | 0%* | 20.74% | 18.81% |
|  | 25-29 | 27.76% | 49.20% | 5.91% | 9.84% | 27.54% | 29.43% | 15.80% | 15.85% | 0.79% | 0.10% | 39.38% | 42.08% | 1.29% | 0.60% | 20.79% | 20.10% |
|  | 30-34 | 43.75% | 45.24% | 10.62% | 8.68% | 30.29% | 32.37% | 15.51% | 12.58% | 1.85% | 0%* | 43.32% | 46.29% | 2.35% | 0%* | 21.85% | 18.74% |
|  | 35-39 | 44.92% | 37.72% | 11.53% | 10.44% | 33.32% | 35.61% | 13.35% | 8.78% | 1.62% | 1.56% | 47.65% | 50.92% | 2.12% | 2.06% | 21.62% | 21.56% |
|  | 40-45 | 40.68% | 27.87% | 15.23% | 9.47% | 36.65% | 39.17% | 8.61% | 6.31% | 5.74% | 2.32% | 52.42% | 56.01% | 6.24% | 2.82% | 25.74% | 22.32% |
|  | 45-49 | 27.86% | 21.42% | 17.18% | 10.76% | 40.32% | 43.08% | 8.03% | 5.84% | 10.34% | 4.85% | 57.66% | 61.61% | 10.84% | 5.35% | 30.34% | 24.85% |
| Cameroon | 15-19 | 0.62% | 2.13% | 1.50% | 1.49% | 22.11% | 23.44% | 0.98% | 2.83% | 1.31% | 1.02% | 31.92% | 33.84% | 1.81% | 1.52% | 21.31% | 21.02% |
|  | 20-24 | 2.54% | 7.48% | 2.03% | 2.60% | 24.32% | 25.79% | 1.92% | 3.75% | 1.62% | 0.79% | 35.11% | 37.23% | 2.12% | 1.29% | 21.62% | 20.79% |
|  | 25-29 | 5.14% | 10.28% | 3.06% | 3.86% | 26.75% | 28.37% | 2.91% | 3.38% | 1.79% | 0.60% | 38.62% | 40.95% | 2.29% | 1.10% | 21.79% | 20.60% |
|  | 30-34 | 8.10% | 9.66% | 3.87% | 3.99% | 29.43% | 31.20% | 2.96% | 2.42% | 1.87% | 0.97% | 42.48% | 45.04% | 2.37% | 1.47% | 21.87% | 20.97% |
|  | 35-39 | 8.60% | 7.26% | 5.25% | 3.67% | 32.37% | 34.32% | 1.84% | 1.93% | 2.53% | 1.76% | 46.73% | 49.55% | 3.03% | 2.26% | 22.53% | 21.76% |
|  | 40-45 | 5.63% | 6.10% | 6.39% | 4.17% | 35.61% | 37.76% | 1.22% | 1.58% | 4.19% | 2.26% | 51.40% | 54.50% | 4.69% | 2.76% | 24.19% | 22.26% |
|  | 45-49 | 3.94% | 5.32% | 6.47% | 4.96% | 39.17% | 41.53% | 1.14% | 1.47% | 5.23% | 2.96% | 56.54% | 59.95% | 5.73% | 3.46% | 25.23% | 22.96% |
| Tanzania | 15-19 | 2.11% | 2.11% | 0.85% | 1.23% | 22.11% | 22.74% | 1.22% | 1.76% | 0.38% | 0.75% | 30.89% | 31.77% | 0.88% | 1.25% | 20.38% | 20.75% |
|  | 20-24 | 4.16% | 6.06% | 1.53% | 2.18% | 24.33% | 25.02% | 1.95% | 2.68% | 0.51% | 0.66% | 33.98% | 34.95% | 1.01% | 1.16% | 20.51% | 20.66% |
|  | 25-29 | 6.87% | 9.52% | 2.52% | 3.53% | 26.76% | 27.52% | 2.34% | 3.48% | 0.68% | 0.91% | 37.38% | 38.44% | 1.18% | 1.41% | 20.68% | 20.91% |
|  | 30-34 | 8.57% | 12.88% | 4.28% | 4.38% | 29.43% | 30.27% | 2.46% | 2.95% | 1.79% | 0.48% | 41.12% | 42.29% | 2.29% | 0.98% | 21.79% | 20.48% |
|  | 35-39 | 9.39% | 11.40% | 5.07% | 4.84% | 32.38% | 33.30% | 3.08% | 2.43% | 2.08% | 1.07% | 45.23% | 46.52% | 2.58% | 1.57% | 22.08% | 21.07% |
|  | 40-45 | 12.33% | 9.88% | 6.39% | 5.49% | 35.62% | 36.63% | 1.60% | 1.34% | 2.08% | 1.93% | 49.76% | 51.17% | 2.58% | 2.43% | 22.08% | 21.93% |
|  | 45-49 | 6.81% | 5.79% | 6.46% | 4.83% | 39.18% | 40.29% | 1.50% | 1.25% | 3.89% | 2.55% | 54.73% | 56.29% | 4.39% | 3.05% | 23.89% | 22.55% |

**Figure S1 : Observed Data vs. Aid Freeze Forecasts.**


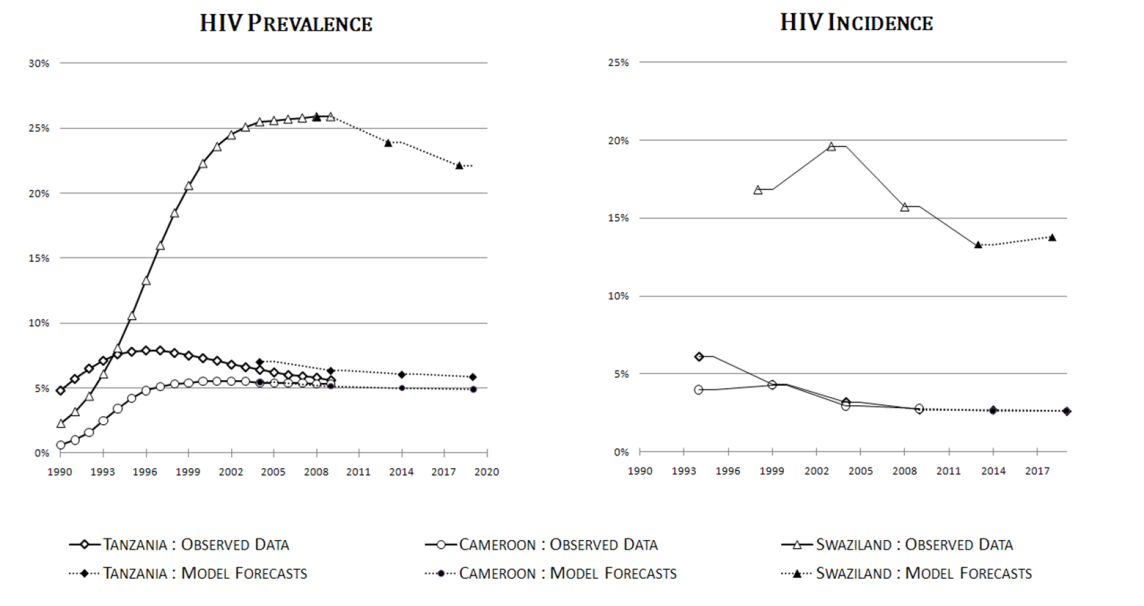

Supplement: Appendix S1 — Detailed methodology of the Micro-simulation Model. (DOCX) [file pone.0034101.s001.docx]
