# Supplementary figures and images for: The Macroeconomic Consequences of Renouncing to Universal Access to Antiretroviral Treatment for HIV in Africa: A Micro-Simulation Model
Source: PLoS One. 2012 Apr 13;7(4):e34101. doi: 10.1371/journal.pone.0034101 (PMC3325986; doi:10.1371/journal.pone.0034101)

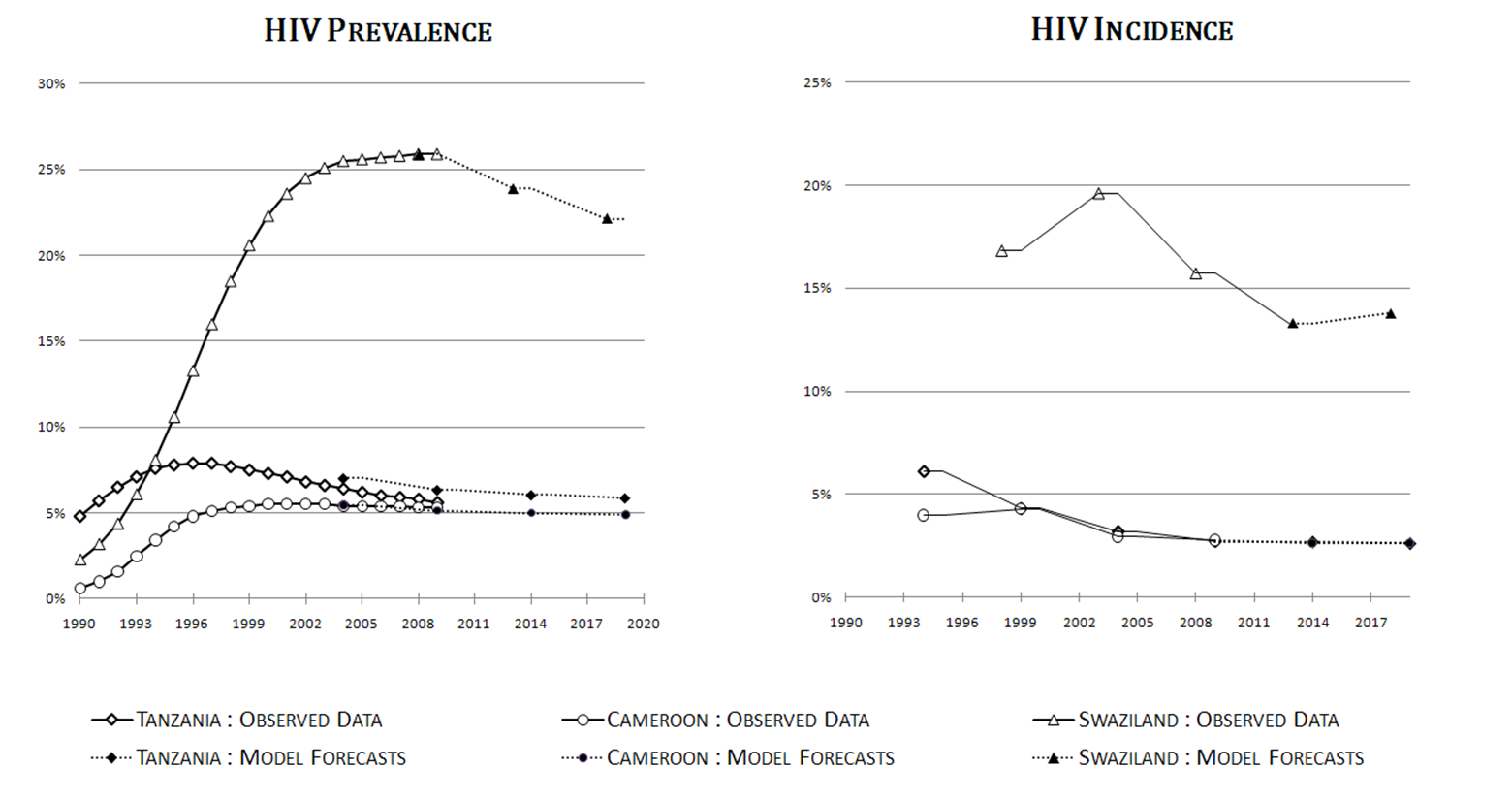

Supplement: Figure S1 — (TIFF) [file pone.0034101.s002.tif]
